# Integrating amyloid and tau imaging with proteomics and genomics in Alzheimer’s disease

**DOI:** 10.1016/j.xcrm.2024.101735

**Published:** 2024-09-17

**Authors:** Gabriele Vilkaite, Jacob Vogel, Niklas Mattsson-Carlgren

**Affiliations:** 1 Department of Clinical Sciences Malmö, SciLifeLab, Lund University, Lund, Sweden; 2 Clinical Memory Research Unit, Department of Clinical Sciences Malmö, Lund University, Lund, Sweden; 3 Department of Neurology, Skåne University Hospital, Lund University, Lund, Sweden; 4 Wallenberg Center for Molecular Medicine, Lund University, Lund, Sweden

## Abstract

Alzheimer’s disease (AD) is the most common neurodegenerative disease and is characterized by the aggregation of β-amyloid (Aβ) and tau in the brain. Breakthroughs in disease-modifying treatments targeting Aβ bring new hope for the management of AD. But to effectively modify and someday even prevent AD, a better understanding is needed of the biological mechanisms that underlie and link Aβ and tau in AD. Developments of high-throughput omics, including genomics, proteomics, and transcriptomics, together with molecular imaging of Aβ and tau with positron emission tomography (PET), allow us to discover and understand the biological pathways that regulate the aggregation and spread of Aβ and tau in living humans. The field of integrated omics and PET studies of Aβ and tau in AD is growing rapidly. We here provide an update of this field, both in terms of biological insights and in terms of future clinical implications of integrated omics-molecular imaging studies.

## Graphical abstract

**Figure:**
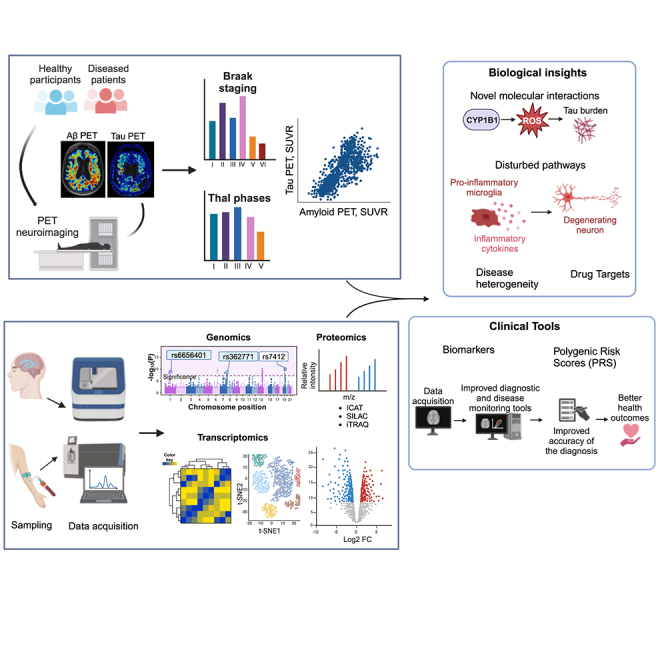

## Teaser

Alzheimer’s disease (AD) is characterized by the aggregation of beta-amyloid and tau in the brain. Vilkaite et al. describe how molecular imaging of these pathologies can be integrated with broad omic strategies, including proteomics and genomics, to achieve novel understanding of disease mechanisms, improve patient management, and identify novel therapeutic strategies.

## Introduction

Alzheimer’s disease (AD) is the most common neurodegenerative disease worldwide. The disease is complex and multifactorial but is characterized by two key pathological changes: the aggregation of abnormally folded β-amyloid (Aβ) peptides in extracellular plaques and hyperphosphorylated tau (p-tau) proteins in intracellular neurofibrillary tangles. Aggregated Aβ and tau can be detected and quantified *in vivo* using positron emission tomography (PET) with tracers sensitive to each pathology (e.g., PiB, florbetapir, and flutemetamol for Aβ,^1^ and flortaucipir, RO-948, and MK-6240 for tau^2^). Aβ and tau aggregation is believed to lead to a cascade of downstream effects, which include atrophy and cognitive decline. Aβ and tau PET have informed on this process, which starts in individuals who are cognitively unimpaired (CU) and continues with mild cognitive impairment (MCI) and dementia. Note that Aβ pathology can be significant in individuals who remain CU during life, while tau pathology has a stronger relationship to clinical symptoms. However, several key issues in the pathophysiology of AD remain unclear. It is not known exactly which pathobiological processes trigger the aggregation of Aβ and tau, or which processes link Aβ and tau together. Factors underlying the heterogeneous patterns of aggregation of (particularly) tau are unclear. Furthermore, although many other pathological processes have been described in AD, e.g., astrogliosis and microgliosis, it is not clear how these are linked to Aβ and tau.

**Figure 1:**
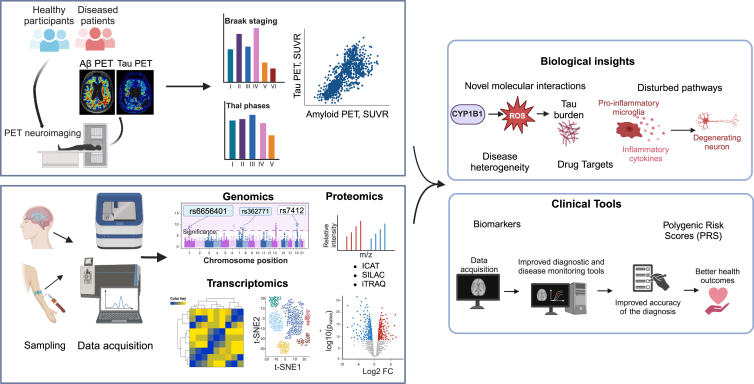
The overall workflow for data generation Top left: shows the typical workflow of generating Aβ and tau PET neuroimaging data. Bottom left: shows the summary of the typical omics data generation workflow. The overall outcomes and results of merging the two workflows together are visualized on the right-hand side of the graph.

We can increase our understanding of how cellular and biochemical functions are linked to the aggregation of Aβ and tau in living humans through the integration of genomics, transcriptomics, and proteomics with molecular imaging of Aβ and tau (Figure 1). In such studies, omics methods may provide broad information on biological mechanisms and risks associated with Aβ and tau pathology. The goal of this review is to give an update on recent discoveries and state-of-the-art strategies in this field. We especially review how integrative omics-PET studies increase our understanding of mechanisms of AD and can help us overcome clinical challenges of diagnosis, prognosis, and personalized treatment.

The focus on integrated omics-PET studies is especially timely now due to a rapid development of high-throughput proteomics methods with high sensitivity and specificity at low sample volumes,^3^ using e.g., mass spectrometry (MS)-based methods, nucleic acid-linked immuno-sandwich assays,^4^ aptamer-based technologies^5^ (covering about 10,000 aptamers with the SomaScan platform), or proximity extension assays^6^ (PEAs, covering over 5,000 proteins with the OLINK HT platform). Genome-wide genotyping, whole-exome sequencing, and whole-genome sequencing are also becoming increasingly available.^4^^,^^7^

Several excellent reviews have been recently published summarizing AD-related changes in transcriptomics, proteomics, and metabolomics, integrating information across diverse sources.^8^^,^^9^^,^^10^^,^^11^^,^^12^^,^^13^ In the present review, we focus on papers that specifically use PET for the measurement of Aβ and tau in humans, restricting our discussion to evidence highly relevant and translatable to the clinical manifestation of AD. We exclude studies that rely only on cerebrospinal fluid (CSF) biomarkers of Aβ and tau. Although CSF Aβ and tau biomarkers generally agree well with Aβ PET for binary classification, and are also associated with tau PET,^14^ CSF biomarkers have limitations in the context of this review. For Aβ, PET offers unique advantages by providing regional information about the spatial distribution of pathology and by offering a greater dynamic range, which makes it possible to track Aβ aggregation in a stage when CSF Aβ biomarkers have plateaued. For tau, CSF AD biomarkers that have been used until now are only moderately associated with tau PET and partly reflect disturbances in soluble tau that are independent of aggregated tau.^15^ For these reasons, we mainly focus here on Aβ and tau by PET rather than CSF, although we acknowledge that PET also has limitations, e.g., high costs and low availability.

## Integrating omics with Aβ PET

The Aβ peptide is formed from the metabolism of amyloid precursor protein (APP).^16^ Aβ peptides are prone to aggregation and form the bulk of the extracellular plaques in AD. Aβ PET can detect and quantify both global and regional Aβ aggregation, with a wide dynamic range from early to late stages of pathology.^17^

### Genome-wide association studies of Aβ PET

Studies integrating genomics with Aβ PET can give information complementary to traditional AD genome-wide association studies (GWASs), which are based on clinical diagnosis. The first such studies emerged soon after the development of Aβ PET, identifying single-nucleotide polymorphisms (SNPs) linked to several genes, e.g., on chromosome 1 (*DHCR24*,^18^ involved in cholesterol synthesis^19^), chromosome 3 (*BCHE*,^20^ a cholinesterase^21^), and chromosome 21 (*APP*^22^). Longitudinal PET studies identified the microglia-related chromosome 3 gene *IL1RAP* as being associated with longitudinal Aβ accumulation.^23^ But these early studies were small, risking false-negative or false-positive results. Sample sizes have increased in new studies, although they remain much smaller than contemporary GWAS for AD as a clinical syndrome. A meta-analysis of three cohorts studied ∼1,000 individuals with PiB and whole-genome genotyping (predicting global cortical standardized uptake volume ratio, SUVR).^24^ As expected, the strongest effect was for the genetic variant defining the *APOE* ε4 allele (rs429358), which is the strongest common genetic risk factor for AD.^25^ There was also a protective effect of the *APOE* ε2 allele, with reduced Aβ burden, in agreement with the known protective role of this allele for AD. Beyond the *APOE* region, 15 genetic variants had suggestive associations with Aβ burden. When analyzing effects conditional on *APOE* ε4, several genetic variants within the *APOE* region remained associated with Aβ burden, suggesting that the *APOE* region had multiple independent signals that may contribute to Aβ build-up. Focusing on the asymptomatic stages of AD, another study included 4,314 CU participants from several cohorts in a GWAS for global cortical PiB and florbetapir quantities.^26^ Strong associations were again seen for the *APOE* region. In contrast to the Yan study, when analyzing the *APOE* region conditioning on *APOE* ε2 and ε4, other variants in the region were no longer significant. This study also suggested that some earlier findings (e.g., *BCHE*) may be cohort-dependent. A novel finding was that variants on chromosome 16, linked to the gene *RBFOX1* (a splicing regulator, associated with psychiatric conditions^27^), were significantly associated with Aβ burden. Orthogonal evidence came from the ROSMAP (The Religious Orders Study and Rush Memory and Aging Project) study, where lower levels of RBFOX1 mRNA in the prefrontal cortex were associated with more Aβ.

A common limitation of these genomic-Aβ PET studies is that they are largely based on participants with European ancestry. A Korean study (*N* = 759) did not find any genome-wide significant associations with Aβ PET beyond *APOE*,^28^ but in gene-based analysis instead of variant-based, six genes were identified (on chromosomes 13, 16, 17, and 21, for genes *LCMT1*, *SCRN2*, *LRRC46*, *MRPL10*, *SP6*, and *OSBPL7*). Another Korean study (*N* = 1,474) instead implicated a variant on chromosome 7.^29^

In what may be the largest study of this kind, 13,409 individuals from 14 cohorts were included.^30^ The study identified, beyond *APOE*, three genes with significant variants (chromosome 1, *CR1*, a complement component receptor, linked to microglial activity^31^; chromosome 14, *FERMT2*, a potential modulator of APP metabolism^32^; chromosome 19, *ABCA7*, linked to cholesterol metabolism^33^). Analysis conditional on *APOE* identified several variants in the *APOE* region with significant associations with Aβ PET, implicating the genes *TOMM40* (linked to mitochondrial function^33^^,^^34^) and *NECTIN2* (encoding an immunoglobulin-like cell adhesion molecule^35^).

Taken together, the results from several GWASs of Aβ PET conducted over more than a decade are informative, but also humbling. In general, findings beyond *APOE* have rarely been replicated between studies. The most recent study by Ali et al. may provide the most solid evidence yet implicating non-*APOE* genes. Potentially, more fine-grained genetic analyses could reveal additional relevant genetic variants. For example, one study utilized RNA sequencing data from human hippocampal tissues in the Allen Human Brain Atlas (AHBA) and the Adult Changes in Thought study and identified exon skipping events associated with AD.^36^ A genetic variant (rs362771) associated with a tendency of exon skipping in the *RELN* gene (potentially linked to phosphorylation and synaptic plasticity^37^) was associated with higher cortical Aβ levels, measured by florbetapir in Alzheimer’s Disease Neuroimaging Initiative (ADNI).

### Polygenic risk scores for Aβ PET

So far, we have discussed associations between individual genetic variants or genes and Aβ load. In contrast, polygenic risk scores (PRSs) aggregate the risk from several genetic factors.^38^ The study by Ali et al. found associations between Aβ PET and a PRS that included all genome-wide significant associations detected in their study.^30^ When used together with age, sex, genetic principal components, and study cohort, the PRS explained 8.7% and 4.7% of the variance in Aβ when including and excluding the *APOE* region, respectively. Another study (*N* = 291 from the INSIGHT cohort) formed a PRS from variants significantly associated with florbetapir PET (used as binary outcome), selected from a set of genetic variants originally associated with clinical AD.^39^ This Aβ-tuned PRS (derived without *APOE* variants) was significantly associated with Aβ positivity in both the original cohort and in ADNI. Gene ontology experiments showed that the PRS was enriched for genes related to Aβ metabolism, protein oligomerization, and cell migration.

Other studies have utilized PRS formed with weights from different clinical AD GWAS. One study^40^ used weights from the Wightman GWAS.^41^ Associations were tested with PiB in the Mayo Clinic Study of Aging (MCSA, *N* = 1,725) and with florbetapir in ADNI (*N* = 1,068), using global SUVR. The PRS explained 8%–9.9% of the variance in Aβ when including the *APOE* region and 1.8%–2.1% when excluding the *APOE* region, in MCSA. In ADNI, the proportion explained was 23.5% when including the *APOE* region, and the non-*APOE* variants uniquely explained 4.4% of the variance in Aβ. In another study from MCSA, an AD PRS (using weights from the study by de Rojas et al.^42^) improved the prediction of PiB positivity compared to a model that used plasma AD biomarkers (e.g., 68% PiB positive by plasma p-tau181, compared to 88% positive by plasma p-tau181 combined with PRS).^43^ A study by Tan et al.^44^ used a related metric, polygenic hazard scores, trained to predict AD age at onset,^45^ with weights from the IGAP (International Genomics of Alzheimer's Project) study.^46^ The score (controlling for *APOE*) was associated with regional florbetapir (*N* = 980 ADNI participants), with global associations and largest effects in frontal regions. Another approach was suggested by Gunter et al., where machine learning models were leveraged to allow for non-linear effects and interactions between variants.^47^ The models were trained with 12 top variants from a large recent AD GWAS,^48^ and the MCSA was used (*N* = 1,791) with prediction of Aβ PiB quantities. Machine learning models improved performance to predict Aβ, e.g., from R^2^ = 0.24 for a standard PRS to R^2^ = 0.28 for a model using XGBoost (based on decision trees; both models also included age, sex, and *APOE* information).

Other studies have analyzed PRS specifically tuned by disease processes. Femminella et al.^49^ used genetic data from IGAP^46^ and built PRS from genome-wide significant variants linked to pathways of immune, endocytotic, or lipid metabolism. Associations were tested between PRSs and both global and regional Aβ SUVR in ADNI (*N* = 709). The lipid-related PRS included variants linked to *CLU* (encoding a multifunctional glycoprotein^50^), *SORL1* (encoding a low-density lipoprotein receptor, suggested to be relevant for APP metabolism^50^^,^^51^), and *ABCA7* and was related to higher widespread Aβ load (although a higher prevalence of *APOE* ε4 in those with high genetic lipid risk could potentially confound the associations).

Several studies have applied PRS to assess the transferability of genetic risks from cohorts largely comprising individuals of European ancestry to other populations. One study with 504 participants in the Japanese ADNI study used genetic effects from the Jansen et al. GWAS^52^ and found associations with PiB (used as negative or positive) also when excluding the *APOE* region.^53^ Another study tested 1,214 Koreans with florbetaben or flutemetamol (also used as negative or positive) and derived PRS from the Kunkle et al. GWAS,^54^ excluding the *APOE* region,^55^ and found significant associations with Aβ positivity. These studies support the transferability of genetic risk effects from individuals with largely European ancestry to Japanese and Korean populations, respectively.

PRS studies have also been applied to longitudinal designs. Luckett et al. studied 90 cognitively healthy individuals with longitudinal flutemetamol^56^ and derived PRS from weights from Kunkle et al.^57^ A PRS including all genome-wide significant variants was associated with continuous longitudinal increase in Aβ, but only when models were also adjusted for the weighted sum of *APOE* ε2 and ε4 alleles (R^2^ = 0.12 compared to R^2^ = 0.09 for the *APOE* variants when used alone).

### Proteomics and transcriptomics studies for Aβ PET

Studies have also integrated transcriptomics or proteomics with Aβ PET, using measurements primarily in blood (superior accessibility) or CSF (potentially more representative of brain changes). The chain of causality is more unclear here compared to genetic studies (i.e., does Aβ lead to altered protein levels, or vice versa?). Instead, these studies can inform about dynamic biological processes that are aligned with the development of Aβ during AD. This is especially relevant to understand changes in biology that occur in early stages, prior to symptoms, when the first changes in Aβ metabolism may occur.

One early study of the plasma proteome and Aβ PET combined a range of proteomics methods (including 2D gel electrophoresis [1,386 proteins assessed] and liquid chromatography-tandem mass spectrometry [LC-MS/MS] [381 protein isoforms from 116 proteins assessed]) to identify large and small proteins associated with global cortical PiB.^58^ The study was conducted in the Baltimore Longitudinal Study of Aging (BLSA) and the AIBL (Australian Imaging, Biomarker, and Lifestyle) cohort and included longitudinal plasma samples from CU individuals, taken up to 12 years prior to PET. Five proteins had consistent associations with Aβ (α2M, albumin, Apo-A1, C3, and haptoglobin). Another study used LC-MS/MS to identify 249 different proteins,^59^ tested associations with CSF biomarkers of AD, and then validated significant proteins with orthogonal techniques (multiplex bead assays and ELISAs) for associations with flutemetamol in an independent cohort. The strongest finding was for increased plasma FCN2 in flutemetamol-positive individuals. Acknowledging differences among previous studies, one attempt was done to reproduce 31 previously identified candidate AD plasma biomarkers in 1,000 participants (both CU and impaired individuals) in the EMIF-AD (European Medical Information Framework—Alzheimer's Disease) project.^60^ Seven proteins (FCN2, B2M, A1AT, apoE, C4, cathepsin D, and CFI) were significantly associated with Aβ status, identified using either CSF Aβ42/Aβ40 or Aβ PET. A classifier using the seven proteins plus age achieved area under the curve (AUC) 0.742 for Aβ positivity (compared to AUC 0.617 for age alone). Another example of a potentially successful plasma proteomic signature is described by Park et al., who developed a multiplex kit for blood biomarkers (including Aβ1-40, ACE, POSTN, and LGALS3BP) to prescreen for PiB positivity.^61^ The proteins were selected based on an earlier MS-based proteomics study^62^ and achieved AUC 0.891 for PiB positivity when used together with *APOE* (compared to AUC 0.783 for *APOE* alone). Plasma proteomics for Aβ PET using aptamer technology has also been attempted. One recent study derived 14 protein-based health indicators, e.g., for cardiovascular risk, kidney function, and other indicators.^63^ The study included 196 non-demented participants from BLSA with PiB (29% PiB positive), but no protein-based health indicator was associated with Aβ PET positivity after correction for multiple comparisons.

Proteomics studies for Aβ PET have also been done in CSF. One paper on autosomal-dominant AD studied 355 AD mutation carriers and 230 non-carriers who underwent PiB, and measurement of 59 CSF proteins by selected reaction monitoring (SRM)-MS.^64^ 33 proteins differed between mutation carriers and non-carriers, with most changing already before symptom onset. Changes in an overall cortical measure of Aβ started about 20 years prior to symptom onset. Sixteen proteins (related e.g., to the matrisome, synapse changes, and stress response) changed before Aβ. In contrast, 17 proteins started to change after Aβ (related e.g., to white matter/axonal integrity, immune activation, and synaptic/neuronal loss). Another study identified 48 from a set of 200 CSF proteins measured by SRM-MS in ADNI, 22 of which differed between patients with AD and controls, and 25 of which were associated with florbetapir with an overall R^2^ = 0.66^65^ (using linear regression with elastic net regularization). Interestingly, the proteins only partly overlapped with proteins associated with CSF Aβ42. The most strongly associated proteins were SMOC1 (SPARC-related modular calcium-binding 1 protein), YWHAZ, and YWHAB (both members of the 14-3-3 family^66^). One recent paper from our group^67^ tested CSF proteins both in relation to Aβ and tau PET and also found associations with SMOC1 for Aβ PET, as described in detail further.

Bridging genomics and proteomics, transcriptomic studies enable *in vivo* studies of gene expression during the continuum of Aβ accumulation. Peripheral expression of 11,727 genes was assessed in cognitively normal individuals in relation to Aβ deposition. 107 genes were differentially expressed in individuals who became Aβ-positive over time, implicating changes in the immune system, protein removal, and metabolism.^68^ One integrative multi-omics study assessing 15,616 transcripts from 94 individuals with Aβ PET suggested a potential blood omics signature for the prediction of Aβ positivity in asymptomatic at-risk subjects, which included three genes among the most discriminant features.^69^ These were *ACSBG2* (involved in lipid metabolism^70^), *RASL11A* (a regulator of rDNA transcription^70^^,^^71^), and *RNU12* (a long noncoding RNA, linked to early-onset cerebellar ataxia^72^).

Analyses of the brain transcriptome can contribute to an understanding of the biological processes that regulate the spread of Aβ. Such analyses can leverage mass-univariate or network-based approaches across thousands of transcripts to compile lists of genes with regional expression patterns similar to a key feature, e.g., the regional expression of Aβ (or tau, as reviewed further) in AD. These gene lists can subsequently be interrogated using gene set enrichment analysis to uncover shared participation in biological processes or molecular components among the compiled genes. Here, we refer to these approaches as “regional transcriptional profiling.” We proposed a staging scheme for Aβ deposition, where regions of the brain were identified that accumulate Aβ early, intermediately, or late in the disease process.^73^ Brain expression of 58,692 transcripts from the AHBA was used to identify genetic pathways that differed between regions involved in different Aβ stages. A cluster of pathways with strong segregation among the regions was associated with voltage-gated ion channel activity, neuropeptide signaling, glutamate signaling, vasodilation, and lipid transportation, suggesting that these processes may be important for the spatiotemporal patterns of Aβ aggregation.

Transcriptomic studies have also zoomed in on links between inflammation and Aβ. One study identified 15 immune-related differentially expressed genes in brain transcriptome data obtained from the AlzData database.^74^ Four of these were differentially expressed in blood in a cohort of 85 individuals who also underwent PET for both Aβ (florbetapir) and microglial activity (TSPO, translocator protein). Only one marker, CD200 (a ligand to a microglia receptor^70^^,^^71^^,^^75^), was significantly (negatively) associated with TSPO PET and was also significantly (negatively) associated with Aβ. Furthermore, CD200 levels partly mediated associations between Aβ and TSPO PET, with regional specificity.

### Omics explaining resilience to Aβ PET

Another class of studies used omics approaches to evaluate if genetic variants moderate the associations between Aβ PET and cognitive decline in AD. These studies operate under the assumption that genetic variants may partly explain that some individuals appear to be more or less severely affected by AD, given their level of Aβ burden. One early study examined 678 ADNI participants for interactions between SNPs and Aβ burden to predict cross-sectional cognition.^76^ Variants on chromosome 12 and 13 were identified, and one of these was validated (including in the ROSMAP neuropathology cohort) and also interacted with Aβ PET to predict cortical thickness. The variant was linked to the gene *IAPP*, which encodes the pancreatic peptide hormone amylin with a role in glycemic control. Another study on 546 Aβ PET-positive individuals (mainly CU) did a GWAS to predict cognitive resilience and identified a variant on chromosome 8, which was validated in ADNI.^77^ The gene implicated was *CNOT7*, a gene linked to synaptic plasticity and hippocampal-dependent learning and memory. One study included several cohorts and identified a genetic variant on chromosome 18 (rs2571244, near the gene *ATP8B1*, critical for maintaining bile acid homeostasis) as modulating cognitive reserve to Aβ load.^78^ A study on 2,953 CU individuals identified 4 genetic variants that moderated the association between Aβ PET and cognition.^79^ One variant (on chromosome 9, near the gene *ARPP21*, involved in dendritic branching^80^) was validated in ADNI, where it interacted with florbetapir SUVR to predict accelerated cognitive decline and atrophy over time.

## Integrating omics with tau PET

The other key AD pathology is tau.^81^ Tau is a microtubule-associated protein, encoded by the *MAPT* gene, and is prevalent in neurons. In AD, there is hyperphosphorylation and accumulation of insoluble intracellular tau protein aggregates, called neurofibrillary tangles, which spread through the brain in a stereotyped pattern.^82^ The spread of tau is associated with atrophy and cognitive decline in AD.^83^^,^^84^

### Genome-wide association studies of tau PET

Study of the relationship between genetic risk factors and tau pathology, as quantified using tau PET (in most cases, using the tracer flortaucipir), is gaining traction in neurodegenerative research. The integration of tau PET with GWAS may give clues to genetic underpinnings that predispose individuals to tau. Ramanan et al. were the first to report a GWAS of tau PET,^85^ specifically in a composite region of interest in the temporal lobes, in a population-based study (*N* = 754 individuals over the age of 50) from the MCSA cohort. Neither *APOE* ϵ2 or ϵ4 alleles, or any SNPs previously reported^46^^,^^54^ to be associated with AD clinical diagnosis, were significantly associated with flortaucipir SUVR. However, two novel SNPs (rs76752255 within *PPP2R2B* [encoding the protein phosphatase 2 regulatory subunit B protein], and rs117402302 near *IGF2BP3* [encoding the insulin-like growth factor 2 mRNA-binding protein 3]) were associated with tau. Guo et al. also performed a GWAS of brain tau load in 543 non-demented participants from ADNI.^86^ They found two SNPs significantly associated with increased tau deposition, rs56298435 (in *ZBTB20*, involved in brain development^87^) and rs150532 (near *EYA4*, encoding a transcription activator with phosphorylation activity). Nho et al. performed potentially the largest tau PET GWAS to date, using 3,136 participants from 12 studies.^88^ This did not replicate previous findings but identified a new SNP associated with tau burden: rs2113389 located between the *RMDN2* (encoding the regulator of microtubule dynamics 2 protein) and *CYP1B1* (encoding the cytochrome P450 family 1 subfamily B member 1 protein) genes, which explained 4.3% of the variance in cortical tau (carriers of the minor allele had greater tau deposition). Besides looking at tau PET GWAS broadly, researchers have also stratified analyses by sex. Wang et al. analyzed 493 participants from ADNI, focusing on genetic variants of 10 genes that have been previously shown to have sex-dependent effects on AD. This study found that *DNAJA2*, *FERMT2*, and *TYW5* were associated with increased tau deposits in women, while three loci within *CR1* were associated with increased deposits in men.^89^

Although the stand-alone studies seem to show convincing results of genetic associations to tau burden, we note that each study has identified and shown different SNPs to be associated, without any SNPs or genes being identified in more than one study.

### Polygenic risk scores for tau PET

Like for Aβ, there have been several studies using PRS to illustrate an individual’s genetic predisposition to tau aggregation. Rubinski et al. derived a PRS using weights from previous GWAS^48^^,^^41^, including 85 AD-related SNPs, and assessed its predictive capability on tau accumulation rates and cognitive decline in 231 participants from ADNI.^90^ This study, while also considering the modulating effects of global Aβ PET, showed that a higher PRS was associated with faster accumulation of cortical flortaucipir SUVR. In another PRS study, Ramanan et al. developed a tau-specific PRS incorporating 14 SNPs associated with flortaucipir SUVR.^91^ This PRS was an indicator of tau deposition (explaining 27.8% of the variance), surpassing the predictive power of Aβ PET (explaining 16.2% of the variance). The PRS was also associated with CSF p-tau levels, postmortem Braak staging, and cognitive decline. Another approach, by Sun et al., investigated PRSs focusing on genetic influences within tau-protein kinases and tau-protein binding pathways.^92^ Their study generated PRSs with and without the inclusion of *APOE*. A higher PRS was associated with elevated global tau levels, even in the absence of *APOE*. Moreover, the PRSs mirrored the spatial tau-spreading pattern across cortical regions, aligning with Braak stages I–VI, although the non-*APOE* PRS was only associated with late-stage tau pathology (Braak stages IV–V). These results were validated through both cross-sectional and longitudinal analyses of CSF tau, suggesting the utility of pathway-specific PRS as a predictor of tau pathology along the AD continuum.

### Studies related to regional susceptibility of tau

A major challenge is to understand why certain regions and cells are more selectively vulnerable or resilient to neurodegenerative pathology in AD. Taking advantage of the fact that tau accumulation is regionally variable, several studies have explored molecular properties of tissue especially susceptible to accruing tau pathology. One such study explored *APOE* ε4 in 350 participants from the Knight ADRC (Alzheimer's Disease Research Center) study with flortaucipir PET and Aβ PET.^93^ *APOE* ε4 carriers showed greater tau accumulation specifically in brain regions showing higher *APOE* gene expression in the AHBA. A study by Zheng et al. examined the ADNI and BioFINDER-1 cohorts with flortaucipir and spatial transcriptomic maps of *MAPT* expression from the AHBA.^94^ This confirmed previous findings that *MAPT* expression has a spatial association with tau PET.^95^ Additionally, a model based on brain connectivity, regional *MAPT* expression, and regional Aβ aggregation offered superior predictions of regional tau PET compared to a model using only brain connectivity. Anand et al. also constructed a tau prediction model from brain connectivity, using ADNI flortaucipir data from 196 AD and MCI participants.^96^ They selected 100 AD-risk genes from previous studies and investigated how the regional expression patterns of these genes related to tau patterns, and to the regions where their connectivity model failed to predict tau levels. Several genes, including *MAPT*, *APOE*, *BIN1*, and *ANK3*, showed expression patterns correlating with regional tau accumulation patterns, while others (e.g., *HS3ST5*) showed negative correlations. An enrichment analysis revealed distinct profiles, implicating different biological pathways for selective vulnerability (e.g., neuronal apoptotic processes, and regulators of cell development and neuronal death) and resilience (e.g., lysosomes, vesicle-mediated transport). A final study explored associations between tau-spreading and high-resolution transcriptomic data with flortaucipir and MRI data of 490 participants from two cohorts, ADNI and HABS (Harvard Aging Brain Study).^97^ Regional transcriptomics data from AHBA were used to study genetic gradients across several brain regions. This identified 577 genes that predisposed the spread of tau, with *APOE* and *SLC1A2* (encoding a glutamate transporter^98^) being the strongest genes implicated. Collectively, these studies suggest that the expression patterns of several AD-risk genes are distributed in a manner consistent with the regional accumulation of tau in AD.

## Integrating Aβ and tau

So far, we have reviewed studies focusing either on Aβ or tau. The canonical hypothesis of AD posits the interaction of these two pathologies as the principal driver of neurodegeneration and cognitive decline. Aβ and tau initiate in different parts of the brain, aggregate along different timelines, and are ostensibly driven (at least initially) by different molecular processes. However, since Aβ and tau pathology often co-occur in patients, disentangling independent molecular associations between the two pathologies is not trivial. To successfully account for both pathologies, creative strategies and/or sophisticated modeling approaches are likely needed.

### Disentangling intrinsic tissue vulnerability to Aβ and tau pathologies

One approach to investigating independent molecular associations with Aβ and tau takes advantage of the fact that these two pathologies are differentially distributed throughout the brain. This observation signals that factors intrinsic to different brain regions may make them differentially vulnerable to the aggregation of Aβ and tau. A trio of studies used Aβ and tau PET as an index of regional vulnerability and compared these vulnerability patterns with regional expression patterns of 58,692 gene transcripts (covering over 20,000 genes) measured using RNA sequencing from the AHBA.^95^^,^^99^^,^^100^ All three studies found a positive spatial association between tau vulnerability and *MAPT* gene expression, while two^99^^,^^100^ found a positive spatial association between Aβ vulnerability and *APP* gene expression. In other words, brain regions expressing higher quantities of transcripts for genes encoding Aβ and tau, respectively, were also more likely to express these two pathologies in AD. Looking across other genome-wide significant AD-risk genes, Sepulcre et al. only found an association between Aβ vulnerability and *CLU* expression. In contrast, Yu et al. found several associations between AD-risk genes and both Aβ (positive: *CNTNAP2*, *MADD*, and *PTK2B*; negative: *INPP5D*, *HLA-DRB1*, and *WWOX*) and tau (positive: *AGRN* and *PLD3*; negative: *ADAMTS4*, *EPHA1*, *CD2AP*, and *PICALM*) spatial maps, which were replicated across two cohorts and, in the case of Aβ, two sets of PET tracers.

All three of the aforementioned studies also enacted data-driven strategies seeking to uncover novel molecular pathways associated with tissue vulnerable to Aβ or tau pathology. Using a regional transcriptomic profiling approach, Grothe et al. and Yu et al. both reported gene sets implicated in response to viral infection and immunity, while Grothe et al. further described pathways relating to different aspects of protein synthesis and mitochondrial respiration (cf. Grothe et al. for an interesting and thorough discussion). Regarding tau, Grothe et al. found that genes with expression patterns positively associated with regional tau-related neurodegeneration tended to be enriched in pathways involving cellular differentiation and neurite formation, extracellular signal-regulated kinase pathways, and proteoglycan metabolism. Digging further into the various genes of interest, Grothe et al. concluded neuroplasticity as a common function, suggesting this as a key attribute of tau-vulnerable regions. Pathways associated with tau vulnerability in the study by Sepulcre et al. and Yu et al. largely replicated similar pathways.

Other more recent studies have continued the theme of using normative transcription patterns from the AHBA to represent intrinsic tissue characteristics, integrating this information with complex multi-factorial models of aging and AD. Building on previous work,^101^ Adewale et al.^102^ built a complex model involving various imaging markers of AD pathology, including Aβ and tau PET. The model used human brain network information to simulate propagation of these pathological markers across the brain, while allowing cortical gene transcript maps to simulate putative local molecular interactions. Fitting this model to imaging data from the ADNI outputted candidate gene-pathology pathways. For example, several genes were suggested as local factors relating to the advancing of tau pathology (*TCAM1*, *NFATC4*, *COASY*, *SACM1L*, *MTF2*, *P4HA2*, *PDHX*, *RAP1GAP*, and *BAD*), Aβ pathology (*HN1L*, *TIPARP*, *FOXJ3*, *TDAP2A*, *STAT3*, and *SDH8*), and the interaction of the two pathologies (*LRP10*, *FAM69A*, *MPC2*, and *NFATC4*). A follow-up study focused specifically on the interaction of Aβ and tau in influencing changes to brain functional activity, this time using a new (and smaller, *N* = 63) dataset.^103^ This study found the Aβ gene list to be enriched for developmental and synaptic terms, the tau list to be enriched for cortical cytoskeletal organization and phosphorylation pathways, and their interaction to be enriched for neuroimmune pathways, especially pertaining to microglial expression.

### Translating multi-omic profiles from neuropathology to *in vivo* PET studies

The aforementioned studies have mostly relied on regional relationships between disparate datasets to form inferences on molecular-pathological interactions. While potentially informative with reference to regional susceptibility, these studies do not offer relationships between molecular pathways and AD neuropathology at the individual level. One approach that researchers have used toward this goal is to develop molecular signatures in postmortem datasets and generalize these signatures to *in vivo* datasets. Iturria-Medina et al. derived a multi-gene signature from prefrontal cortex bulk transcriptome data (39,579 probes) associated with increasing Aβ and tau neuropathology and cognitive decline.^104^ The same approach was applied to the blood transcriptome from living patients, and a signature once again emerged that was elevated in people with PET evidence of Aβ and tau pathology. Only a small number of genes contributed to both the *ex vivo*- and *in*-*vivo*-derived signatures, but the gene lists nonetheless showed some overlap in functional enrichment. Beta-1 adrenergic receptor signaling, G protein signaling, and blood coagulation were terms strongly enriched among genes from both signatures, as well as other terms relating to immune response, WNT signaling, and angiogenesis.

Another group utilized a combination of genetics and single-cell transcriptomics to observe cell-type-specific influences on Aβ and tau pathology.^105^ Genes with neural cell-type-specific signatures were derived from the prefrontal cortex of individuals with limited neuropathology, and cell-type specific PRSs for AD were derived using only SNPs within 30 MB of these genes. In a postmortem dataset, the astrocyte PRS was specifically associated with Aβ, while the astrocyte, microglial, oligodendrocyte, and excitatory neuron PRS were all associated with tau pathology, with the microglia PRS having the strongest association. These patterns were replicated to some degree in a large *in vivo* dataset with both genetic information and Aβ and tau PET. In a sample of nearly 3,000 individuals, cortical florbetapir SUVR was associated with astrocyte, oligodendrocyte, and especially microglial PRS, while in a smaller set of 300 individuals, only the microglial PRS related to flortaucipir SUVR in the temporal lobes. These studies together suggest some promise in the approach of transferring information across different omic modalities.

### Differential proteomic associations between Aβ and tau PET

One of the most exciting frontiers in neuroscience is the high-throughput measurement of thousands of proteins from the CSF of living humans. While the last two years have seen an explosion of AD proteomics studies,^106^^,^^107^^,^^108^ very few have examined changes associated with aggregated Aβ and tau pathology as measured with PET. For example, Karlsson et al.^109^ surveyed 2,944 proteins in search of “reference proteins” that enhance the precision of CSF biomarkers for tau pathology by accounting for individual-specific physiology. This approach showed that CBLN4, PTPRN2, and PTPRS enhanced the correlation between CSF p-tau181 and tau PET SUVR, while NTRK2, NTRK3, and BLMH additionally enhanced the correlation between CSF Aβ42/40 ratio and Aβ-PET. While these proteins are not relevant to neuropathology, they may relate to processes relevant to the metabolism of Aβ or tau in physiological conditions, making them important candidates for further study.

Pichet Binette et al.^67^ recently characterized shared and unique proteomic changes associated with aggregated Aβ and tau pathology, using PEA for 1,331 proteins in the BioFINDER cohorts and finding a core set of 15 proteins associated with both pathologies. Proteins associated with Aβ pathology were mainly expressed in glial cells and enriched for functions relating to protein ubiquitination, cellular detoxification, oxidative stress, and mitochondrial processes. One of the strongest Aβ-associated proteins, SMOC1, was additionally associated with Aβ PET SUVR even when adjusting for tau levels, and follow-up immunohistochemistry analysis showed SMOC1 colocalization with Aβ plaques in AD brains. Meanwhile, the presence of both Aβ and tau pathology resulted in upregulation of proteins with functions enriched for nucleotide metabolism. The abundance of several proteins was elevated in people with faster longitudinal tau accumulation among Aβ-positive individuals, including FAPB3, ENO1, ENO2, YWHAQ, NRGN, MAPT, TMSB10, TBCA, RWDD1, and DDT. These findings are promising and are of great interest to the community, but additional proteomics studies measuring both Aβ and tau PET will be needed to establish the replicability of these findings.

## New insights from integration of omics and PET

**Figure 2:**
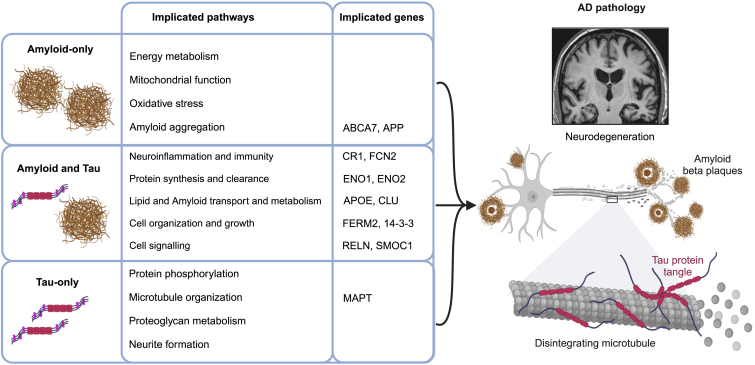
Overview of the pathways, proteins, and genes implicated in AD pathology The pathways, proteins, and genes are categorized by their associations with Aβ PET and Tau PET markers in the studies discussed throughout the review.

The studies reviewed here integrate rich genomics and proteomics data with molecular imaging of Aβ and tau to track the spatiotemporal development of AD pathology *in vivo*. Table 1 lists genes and pathways described across multiple papers, further summarized in Figure 2.

**Table 1:** Genes, proteins, and pathways associated with Aβ and tau PET

Genes and proteins	SNP	Pathways	Ref number	Aβ or Tau PET	Research techniques	Direction of association
ABCA7	rs12151021	lipid transport, metabolism, Aβ processing	Ali et al.; Femminella et al.^30^^,^^49^	Aβ	GWAS, pathway-specific PRS	Positive association
APP; Aβ1-40		neuron death, neuron apoptotic process	Park et al; Grothe et al.; Yu et al.^61^^,^^99^^,^^100^	Aβ	Plasma proteomics, regional transcriptomic profiling	Positive association
CR1	rs6656401	immune receptor activity, cytokine binding	Ali et al.; Wanget al.^30^^,^^89^	Aβ	GWAS	Positive association
FCN2		innate immunity	Westwood et al.; Westwood et al.^59^^,^^60^	Aβ	Plasma proteomics	Positive association
SMOC1		calcium-ion binding, PI3K-Akt signaling pathway, cell growth	Johnson et al.; Haque et al.; Pichet Binette et al.^64^^,^^65^^,^^67^	Aβ	CSF proteomics	Positive association
		abeta aggregation	Xicota et al.; Luckett et al.^39^^,^^68^	Aβ	Whole-blood transcriptomics, optimized PRS	Positive association
		cellular signaling	Mattsson et al.; Grothe et al.; and Sanchez-Rodriguez et al.^73^^,^^99^^,^^103^	Aβ	Regional transcriptomic profiling, CSF proteomics	Positive association
		energy metabolism	Johnson and Luckett et al.^64^^,^^68^	Aβ	CSF proteomics, whole-blood transcriptomics	Positive association
		mitochondrial respiration	Pichet Binette and Grothe et al.^67^^,^^99^	Aβ	Regional transcriptomic profiling, CSF proteomics	Both, positive and negative associations
		oxidative stress and detoxification	Johnson et al.; Pichet Binette et al.^64^^,^^67^	Aβ	CSF proteomics	Positive association
APOE	rs7412 rs429358	metabolism and transport of cholesterol and lipids	Ramanan et al.; Yan et al.: Westwood et al.; Dincer et al.; Montal et al.^20^^,^^24^^,^^60^^,^^93^^,^^97^	Aβ, Tau	Regional transcriptomic profiling, plasma proteomics, GWAS	Both, positive and negative∗ associations
CLU		promote cell survival, lipid metabolism and transport	Femminella et al.; Sepulcre et al.; Sanchez-Rodriguez et al.^49^^,^^95^^,^^103^	Aβ, Tau	Pathway-specific PRS, regional transcriptomic profiling	Positive association
ENO1		Glycolytic pathway, energy production	Johnson et al.; Pichet Binette et al.^64^^,^^67^	Aβ, Tau	CSF proteomics	Positive association
ENO2		Glycolytic pathway, energy production	Johnson et al.; Pichet Binette et al.^64^^,^^67^	Aβ, Tau	CSF proteomics	Positive association
FERMT2	rs117834516	Cell adhesion and shape, axonal growth, plasticity	Ali et al.; Wang et al.^30^^,^^89^	Aβ, Tau	GWAS	Positive association
RELN	rs362771, COLBOS	Neuronal migration and development, cell-cell interactions	Han et al.; Lopera et al.^36^^,^^110^	Aβ, Tau	Transcriptomics, whole-genome sequencing (WGS), whole-exome sequencing (WES)	Negative association
YWHAB		Protein kinase inhibitor activity, protein binding, cell cycle, apoptosis, long-term potentiation	Haque et al.^65^, Johnson et al.^64^	Aβ, Tau	CSF proteomics	Positive association
YWHAZ		RNA binding, protein kinase binding, MAPK and PI3K-Akt signaling pathways	Haque et al.^65^, Johnson et al.^64^	Aβ, Tau	CSF proteomics	Positive association
		Aβ metabolism	Xicota et al.; Anand et al.^39^^,^^96^	Aβ, Tau	Regional transcriptomic profiling, optimized PRS	Both, positive and negative associations
		Cytoskeletal organization	Pichet Binette et al.; Yu et al.; Sanchez-Rodriguez et al.^67^^,^^100^^,^^103^	Aβ, Tau	Regional transcriptomic profiling, CSF proteomics	Positive association
		Lipid metabolism and transport	Femminella et al.; Mattsson et al.; Sepulcre et al.^49^^,^^73^^,^^95^	Aβ, Tau	CSF proteomics; regional transcriptomic profiling, pathway-specific PRS	Positive association
		Neuroimmunity	Johnsona et al.; Luckett et al.; Anand et al.; Grothe et al.; Yu et al.; Sanchez-Rodriguez et al.^64^^,^^68^^,^^96^^,^^99^^,^^100^^,^^103^	Aβ, Tau	CSF proteomics, whole-blood transcriptomics, regional transcriptomic profiling	Both, positive and negative associations
		Neuroinflammation	Anand et al.; Sanchez-Rodriguez et al.^96^^,^^103^	Aβ, Tau	Regional transcriptomic profiling	Positive association
		Neuronal death	Johnsona et al.; Anand et al. ^64^^,^^96^	Aβ, Tau	CSF proteomics, regional transcriptomic profiling	Positive association
		Protein removal	Pichet Binette et al.; Luckett et al.; Anand et al.^67^^,^^68^^,^^96^	Aβ, Tau	Whole-blood transcriptomics, regional transcriptomic profiling, CSF proteomics	Positive association
		Protein synthesis	Grothe et al.; and Yu et al.^99^^,^^100^	Aβ, Tau	Regional transcriptomic profiling	Negative association
		Synaptic reorganization	Johnson et al.; Yu et al.; Sanchez-Rodriguez et al.^64^^,^^100^^,^^103^	Aβ, Tau	Regional transcriptomic profiling, CSF proteomics	Positive association
		Development	Sepulcre et al.; Grothe et al.; Sanchez-Rodriguez et al.^95^^,^^99^^,^^103^	Aβ, Tau	Regional transcriptomic profiling	Positive association
MAPT		Microtubule organization, MAPK signaling	Pichet Binette et al.; Zheng et al.; Sepulcre et al.; Grothe et al.^67^^,^^94^^,^^95^^,^^99^	Tau	CSF proteomics, regional transcriptomic profiling	Positive association
		Neurite formation	Sepulcre et al.; Grothe et al.^95^^,^^99^	Tau	Regional transcriptomic profiling	Positive association
		Protein phosphorylation	Sun et al.; Anand et al.; Sanchez-Rodriguez et al.^92^^,^^96^^,^^103^	Tau	Regional transcriptomic profiling, pathway-specific PRS	Positive association
		Proteoglycan metabolism	Sepulcre et al.; Grothe et al.^95^^,^^99^	Tau	Regional transcriptomic profiling	Positive association

The table summarizes individual proteins, genes, genetic variants, or biological pathways that have been linked to Aβ PET, tau PET, or both, in two or more papers reviewed here. ∗APOE rs7412 (APOE e2) is a genetic factor associated with decreased risk of AD.

The genes identified though Aβ-GWAS inform on disease mechanisms that may increase the propensity for Aβ accumulation, particularly implicating genes related to microglial activity and lipid homeostasis. The CSF proteomics studies suggest that the first changes in Aβ PET occur in parallel with subtle changes in brain metabolism and cellular stress, but before downstream effects on inflammation and overt injury. One protein repeatedly associated with Aβ aggregation is SMOC1, a matrisomal protein enriched in glial cells (specifically oligodendrocyte precursor cells), with an unclear function in AD, but strongly associated with Aβ plaques in neuropathology studies.^111^

Studies integrating tau PET with omics are more scarce and smaller than corresponding studies for Aβ-PET (Table 1; Figure 2), due to the more recent development of tau PET. CYP1B1 and RELN have emerged as potential genes of interest. Across studies, tau-associated regions showed higher expression of genes relating to neurite formation and proteoglycan metabolism, processes that may point to greater levels of synaptic plasticity, and perhaps to regions involved in memory. These findings agree with a growing body of literature that supports the notion of specific excitatory cell populations with additional shared qualities (e.g., high in neurofilament, low in calcium-binding protein, weakly myelinated) that have an enhanced susceptibility to tau tangle aggregation.^112^^,^^113^

Certain biological pathways emerged in association with both Aβ and tau (Table 1; Figure 2), perhaps the most common being neuroimmunity and neuroinflammation. This theme corresponds well to the finding that many genome-wide significant genes associated with AD are expressed in microglia,^114^ the brain’s resident immune cell. Astrocytic and microglial inflammatory processes are common and perhaps ubiquitous features of the AD pathophysiological process.^115^ Many studies seek to establish whether the role of neuroinflammation is causal to AD progression, with one hypothesis suggesting it as an element bridging Aβ and tau and/or catalyzing the emergent toxicity of their interaction.^116^^,^^117^^,^^118^ Studies reviewed here support the notion that neuroinflammation is involved in the response to both Aβ and tau accumulation, though they do not resolve whether this response is protective, harmful, or complex. Other common pathways between Aβ and tau included protein removal and synthesis, lipid metabolism and transport, synaptic organization, and cell death. These processes likely reflect the evolving response of neurons to pathological stress, though specific proteins driving these changes remain to be identified.

## Promise and potential of translation to clinical utilities

One of the ultimate goals of the studies reviewed earlier is discovering insights into Aβ and tau biology that may result in clinical applications (Figure 1). Such applications may include improving AD diagnosis and prognosis or guiding development and use of new therapies. Genomic studies have been useful in the construction of PRSs that quantify risk for AD. A potential use case of such a strategy might include enrichment of patients using a tau-derived PRS, which may reduce sample sizes for clinical trials using tau PET as the outcome.^41^^,^^90^ Proteomics studies also show great clinical potential. Prior research has shown that certain proteins, individually or in combination, can be used as highly specific markers of disease processes. As a proof of concept, excellent biomarkers of Aβ and tau pathology are already well established as diagnostic markers and markers of pathological change.^119^ The translation of these biomarkers to blood plasma positions them on the precipice of transforming the clinical landscape of AD.^120^ Studies reviewed here show examples of how even these well-known biomarkers can be improved through careful profiling of proteomic data.^109^ However, there is also still much work to be done in developing proteomic markers that can reliably capture processes upstream or downstream to Aβ or tau deposition, which may be helpful for monitoring treatment effects or disease progression.

## Limitations and future directions

Although the studies reviewed here have made considerable contributions to our understanding of the development and spread of Aβ and tau in living humans, we also note several limitations. In general, many studies remain quite small and may be underpowered to detect associations, e.g., for individual genetic variants being associated with Aβ or tau PET. We note that reproducibility of individual genetic variants or implicated genes is rare, although some patterns emerge with common biological pathways providing reproducibility at a system level (Table 1 and Figure 2). Fortunately, the sample sizes in integrated PET-GWAS are increasing rapidly. As larger cohorts become available, we expect these studies to reveal additional genetic variants associated with Aβ or tau PET burden, solidify true positive findings, and rule out previous false-positive findings. However, we also note that as larger studies are needed to detect effects, the influence of the identified genes will also be smaller and will likely only have a minor effect on the overall pathology. Future explorations of AD genomics may require more in-depth exploration of rare and structural variants, which can be achieved through whole-genome sequencing efforts.^121^^,^^122^^,^^123^^,^^124^ Additionally, analytic frameworks more suited for modeling gene-by-environment interactions will likely be necessary and can be complemented by advances in epigenomics.^125^^,^^126^^,^^127^^,^^128^ Quantitative trait loci studies may also boost power, precision, and confidence for detecting functional variants dynamically relevant to AD and AD-related processes.^129^

The Aβ PET-PRS studies demonstrate that polygenic effects can be harnessed to partly predict Aβ load, but the additional explained variance when incorporating effects beyond the *APOE* region is mild to moderate. This is similar to PRS for AD in general and is related to the fact that a large proportion of the heritability of AD still remains unaccounted for.^130^ The added value of a polygenic assessment to predict Aβ PET for clinical purposes, compared to only *APOE*, or perhaps *APOE* and a few other key genetic factors, therefore remains questionable. We also note that most PRS studies reviewed here rely on clumping and thresholding of effects at specific strengths of significance (e.g., deriving a PRS from all genetic variants that achieved genome-wide significant effects, *p* < 5∗10^−8^), although other methods to derive PRS could potentially have greater power to detect significant effects.^131^

We reviewed several studies using regional transcriptomic profiling to better understand molecular properties of tissue susceptible or resistant to Aβ and tau pathology. These studies provide an exciting synergy between imaging and whole-brain transcriptomic data, but their value remains questionable in the face of several important limitations. Nearly all the studies reviewed used the AHBA for transcriptomic profiling—a dataset composed of only six individuals—and ADNI to extract regional Aβ and tau distributions. Despite using the same datasets, results varied between studies, likely related to how each study processes, analyzes, and interprets such high-dimensional transcriptomic data. Such data are notoriously susceptible to methodological differences^132^ and suffers from substantial autocorrelation^133^ that is challenging to address.^134^ Several studies reviewed here showed an overlap between e.g., regional tau PET and intrinsic regional *MAPT* expression (Table 1), giving a solid proof of principle for such studies to identify disease-relevant genes or pathways. However, such studies usually result in sets of hundreds of genes, where findings that are relevant to AD create something of a “needle-in-the-haystack” conundrum. The process of functional enrichment of these gene sets also suffers from important biases,^135^ related to incomplete annotation and unknown pleiotropic effects. Finally, and perhaps most pressingly, spatial overlap between transcriptomic signals and Aβ or tau is not necessarily indicative of any association between these elements. None of the studies reviewed engaged in functional validation to confirm associations. However, other studies have shown such validation of computational transcriptomics is possible and can provide crucial insights.^136^ These studies do have unique value in helping to understand the pillars of brain organization that may influence the development of AD in humans and will benefit from emerging high-resolution spatial transcriptomics studies.

Proteomics studies show great promise, but are not without their own pitfalls. Thus far, PET-omic studies have not taken sufficient advantage of the unique ability of PET to capture fine-grained measures of regional pathological burden. We remain in the early days of high-throughput proteomics, and other limitations will no doubt be discovered. As an example, early work has already shown that individual differences in protein levels can create spurious correlations among proteins.^109^ However, the ceiling remains high for high-throughput proteomics in AD, with exciting and important new findings likely on the horizon.

## Acknowledgments

We are grateful to Alexa Pichet Binette for discussions about this review.

The authors are supported by the SciLifeLab & Wallenberg Data Driven Life Science Program (grant: KAW 2020.0239), the Swedish Alzheimer Foundation (AF-994229 and AF-994626), the Swedish federal government under the ALF agreement (2022-Projekt0107), the Skåne University Hospital Foundation, Regionalt Forskningsstöd, the 10.13039/501100006129Konung Gustaf V:s och Drottning Victorias Frimurarestiftelse, Familjen Rönnströms Stiftelse (FRS-0003), WASP and DDLS Joint call for research projects (WASP/DDLS22-066), the Swedish Brain Foundation (FO2023-0163), the 10.13039/501100004359Swedish Research Council (2021-02219), and the Crafoord Foundation (20230790). Figures were created with BioRender.com.

## Declaration of interests

N.M.-C. has a consulting agreement with Biogen. The other authors have no disclosures to report.
